# The Ability of Vaping Technology to Deliver an Equivalent Respirable Dose of Beclomethasone Dipropionate Compared to Nebulization

**DOI:** 10.3390/pharmaceutics16111396

**Published:** 2024-10-30

**Authors:** Cyrille Bruneau, Clément Mercier, Lara Leclerc, Jérémie Pourchez

**Affiliations:** Mines Saint-Etienne, Université Jean Monnet, INSERM, U 1059 Sainbiose, Centre CIS, F-42023 Saint-Etienne, France; cyrille.bruneau@etu.emse.fr (C.B.); clement.mercier@emse.fr (C.M.); leclerc@emse.fr (L.L.)

**Keywords:** vaping drug delivery system, jet nebulizer, respirable dose, aerosol therapy, chronic respiratory diseases, beclomethasone dipropionate

## Abstract

**Background/Objectives**: This study focuses on the ability of vaping technology to deliver beclomethasone dipropionate compared to nebulization. **Methods**: An in vitro comparison of aerosol properties in terms of respirable dose with the Glass Twin Impinger and the mass median aerodynamic diameter using the Next Generation Impactor was performed. The respirable dose delivered in a vaping drug delivery system (VDDS) puff as a function of concentration was quantified by high-pressure liquid chromatography coupled with an ultraviolet detector. **Results**: The mass of drug contained in a single puff of 55 mL of aerosol varied between 0.94 and 1.95 µg for a refill liquid concentration range of 400 to 1600 µg/mL. The analysis of the particle size distribution shows an advantage for a VDDS in producing smaller particles compared to nebulization (1.56 ± 0.05 µm vs. 2.30 ± 0.19 µm). In total, 81 puffs are needed to reach the dose equivalent to nebulized beclomethasone dipropionate under these specific experimental conditions, which corresponds to an aerosol duration of about 4 min (i.e., four times lower than the jet nebulizer) and a patient administration time of about 45 min (i.e., three times higher than the jet nebulizer). **Conclusions**: The results show the potential of vaping devices as an alternative to nebulizers for the administration of beclomethasone dipropionate in an equivalent respirable dose.

## 1. Introduction

Chronic respiratory diseases (CRDs) affected more than 454 million people and were responsible of 4 million deaths worldwide in 2019 [1]. They include various pathologies such as asthma or chronic obstructive pulmonary disease (COPD), which is the third leading cause of death worldwide after cardiovascular diseases [2,3]. These two pathologies are characterized by breathing problems due to airway obstruction. The causes of these diseases are different, but their pathophysiology shares some similarities such as hyperinflammation of the airways causing damage and overproduction of mucus responsible for airflow limitation [4,5]. CRDs are non-communicable diseases that cannot be cured, so existing treatments consist only of relieving symptoms, preventing worsening, and improving daily life with several medications such as bronchodilators or inhaled corticosteroids as daily treatment or during crises [6].

The administration of these drugs by inhalation, known as aerosol therapy, is favored because it has only a local effect and fewer systemic side effects but requires medical equipment to produce aerosols [7]. Aerosol therapy has evolved considerably since its beginnings several millennia ago, and there are currently different types of devices such as pressurized metered-dose inhalers (PMDIs), dry powder inhalers (DPIs) or nebulizers [8,9]. Nebulizers are not the most commonly used devices on the market but have the advantage of being suitable for most medications and patient types [10]. Even within this family of devices, there are different technologies for generating the aerosol [11]. However, the main problems of nebulizers are their size, which forces patients to use them at home, the duration of nebulization, and poor drug delivery efficiency to the lungs [10,11,12]. Therefore, it is important to develop innovative technologies to improve the use and efficiency of aerosol therapy without high costs. A vaping drug delivery system (VDDS) could be an alternative for medicine delivery by the inhaled route [13]. These vaping devices generally used to deliver nicotine are composed of at least three parts: a battery, a heating coil, and an atomizer [14]. The inhaled aerosol is produced by the vaporization of the e-liquid in contact with the heating coil. The increasing use of these devices urges the market to constantly evolve and create new designs of aerosol technologies [15]. Although research around drug vaping is still at early stages, some researchers have demonstrated an interest to keep going further [16,17,18,19,20,21,22]. Our team has thoroughly investigated the feasibility of administering terbutaline sulphate, a bronchodilator, via a VDDS. A VDDS was very efficient in generating submicron carrier droplets containing drug molecules at a constant drug concentration [18]. Interestingly, when the VDDS was used up to a power of 40 W, no thermal degradation of terbutaline was observed and the mass median aerodynamic diameter (MMAD) remained identical to that of a jet nebulizer under clinical conditions [18]. In addition, the delivery of terbutaline with the VDDS was highly dependent on the technical parameters of the VDDS, including resistance, power, and the nebulizer, to achieve a similar respirable dose and MMAD compared to conventional nebulization [19]. In a recent study, a VDDS was found to be unsuitable for the delivery of other bronchodilators (ipratropium and salbutamol), mainly due to thermal degradation of the drugs [20], while other studies successfully delivered salt-free salbutamol with a VDDS over a power range of 20–40 W [22] or fluticasone propionate with different vaping devices [21]. Overall, the above studies emphasize the potential of a VDDS for efficient delivery of inhaled drugs under appropriate physicochemical and technical parameters.

This study focuses on the class of inhaled corticosteroids, and in particular beclomethasone dipropionate, which is commonly used to treat asthma and COPD [23,24,25]. Several parameters of the nebulization of beclomethasone dipropionate (respirable dose, duration, and aerodynamic size distribution) were evaluated in vitro and then compared to the delivery of the drug by an innovative technology from a VDDS. This alternative could increase interest in the production of airborne drugs to improve the quality of life of patients.

## 2. Materials and Methods

### 2.1. E-Liquid Formulation

The e-liquid was prepared using beclomethasone dipropionate (BDP) powder (PHR1619 Supelco, Sigma Aldrich, St. Louis, MO, USA) and mixed with Poloxamer 188 (P188) (Pluronic^®^ F-68 biochemica, panreac applichem, Darmstadt, Germany) as a surfactant in a ratio of 2:1 (*w*/*w*) BDP:P188 in deionized water. The study focused on 4 different concentrations: 400 µg/mL, 800 µg/mL, 1200 µg/mL, and 1600 µg/mL. For the concentration of 400 µg/mL, a stock suspension of 2 mg/mL was diluted in a *v*/*v* ratio of 87.5% PDO (1,3-propanediol) (Vegetol^®^ from Xeres laboratory, Le Blanc, France) and 12.5% BDP suspension. For the other concentrations, the e-liquid was prepared directly by adding the beclomethasone dipropionate powder to a solution of P188, deionized water, and PDO at the *v*/*v* ratio of 87.5% PDO and 12.5% water with mixing until homogenization. The PDO-based formulation was preferred over a mixture of propylene glycol and glycerol, which is normally used for the formulation of e-liquids. This choice was justified by the comparable aerodynamic properties and a lower thermal degradation of PDO compared to propylene glycol and glycerol [26]. The quantity of materials used and the dilutions made for the homemade preparations can be found in Table 1.

### 2.2. Respirable Dose Fraction

**Figure 1:**
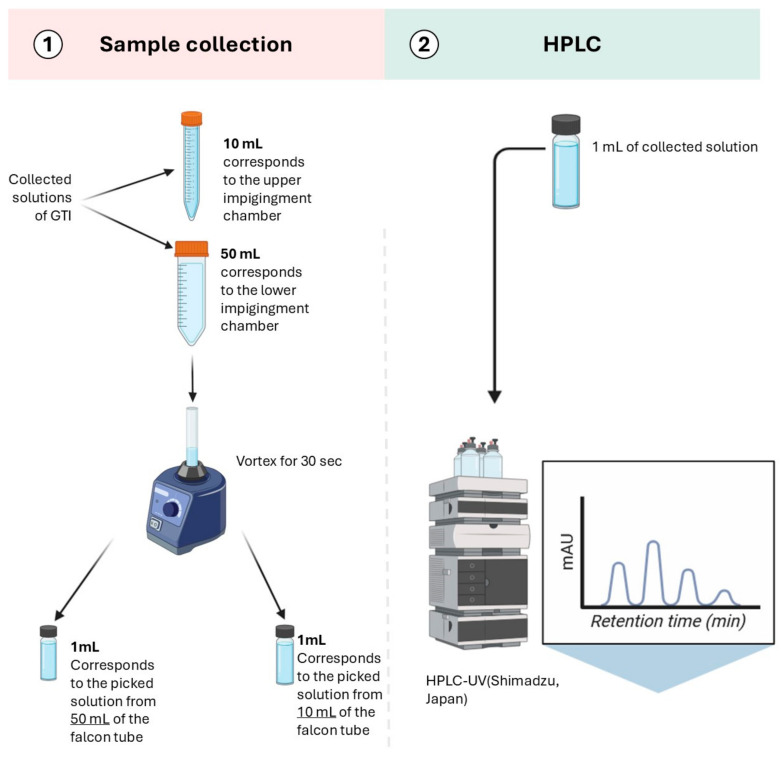
Preparation and HPLC–UV analysis of samples in upper and lower chambers of GTI. Reproduced with permission from Mariam C, Int. J. Pharm. 2022 [19].

The respirable dose fractions were collected using a Glass Twin Impinger (GTI) (Copley Scientific, Colwick, UK) to separate the particles by size according to the monograph of the European Pharmacopoeia [27]. This device represents the airway and is designed to separate the particles into an upper chamber and a lower chamber containing the respirable dose fraction with a cut-off at 6.4 µm. The GTI was connected to a vacuum pump (model LCP5, Copley Scientific, Colwick, UK) set at 60 ± 5 L/min. The solvent used in the chambers was HPLC-grade acetonitrile from Fisher Scientific. Due to the evaporation of acetonitrile during the tests, the volume in the chambers of the GTI was checked every 2 min or between each series for the VDDS and topped up if necessary. The contents of the chambers were then diluted in acetonitrile to give known volumes of 10 mL and 50 mL for the upper and lower chambers, respectively. Then, 1 mL from each chamber was sampled into a 1.5 mL vial (ND9, VWR) for high-pressure liquid chromatography (HPLC) to quantify the drug mass (Figure 1).

### 2.3. Aerodynamic Size Distribution

The particles contained in the aerosol are deposited differently in the airways depending on their aerodynamic size. In order to compare the hypothetical deposition in the lungs, the mass median aerodynamic diameter (MMAD) and the geometric standard deviation (GSD) were determined using a Next Generation Impactor (NGI) (Copley Scientific, Colwick, UK). This impactor consists of 8 stages and sorts the particles according to different cut-off values depending on the aspiration flow. The flow rate of the vacuum pump was set to 15 L/min to compare the nebulizer and VDDS. Deposits from each stage were rinsed with 2 mL acetonitrile, and 1 mL was then transferred to a 1.5 mL vial for HPLC–UV analysis. MMAD, GSD, and statistical t-test calculations were then performed using Microsoft Excel software (Version 2409).

### 2.4. Generation of Aerosols

**Figure A1:**
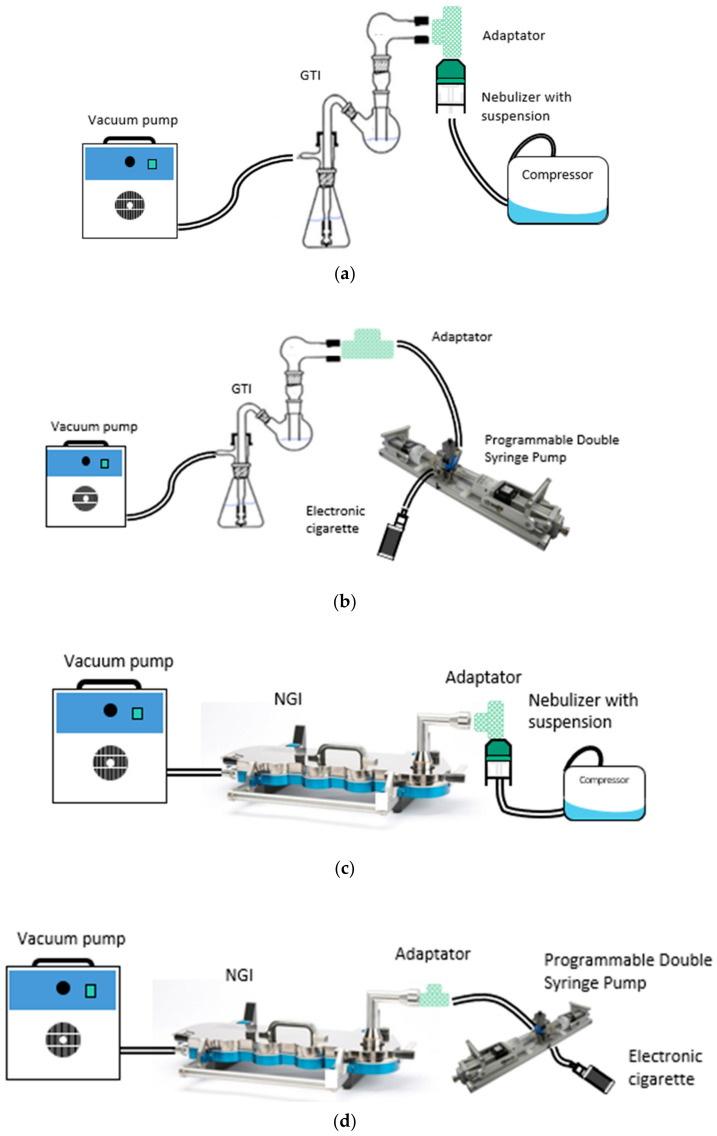
(**a**). Set-up schema for generation of aerosol by nebulizer and collection within GTI. (**b**). Set-up schema of aerosol generation with vaping device and PDSP and collection within GTI. Schema of GTI Reproduced with permission from Mariam C, Pharm. Res 2022 [18] and modified. (**c**). Set-up schema for collecting aerosol by nebulizer with NGI. (**d**). Set-up schema for collecting aerosol by vaping device and PDSP with NGI.

The study compared two different devices, a jet nebulizer (Cirrus™2, Intersurgical, Croissy-Beaubourg, France) and a vaping device (VDDS, istick TC40W coupled with a GS-Tank atomizer, Eleaf, Amersfoort, The Netherlands), both of which produce an aerosol for the inhalation of medication. The nebulizer was filled with 2 mL Beclospin^®^ 800 µg/2 mL (Chiesi, Bois-Colombes, France) and operated until the end of aerosol generation. To generate the aerosol with the VDDS, the tank (GS-Tank atomizer, Eleaf) was filled with 2.5 mL of homemade e-liquid and fitted with a configurable battery (istick TC40W, Eleaf). The tests were conducted with fixed parameters for the wattage (30 W) and resistance (1.5 Ω) (GS Air, Eleaf). The VDDS was fully charged before each test. The puffs were generated using a modular puffing machine (Programmable Dual Syringe Pump PDSP^®^, Burghart Messtechnik^®^, Holm, Germany) connected to the vaping device and the GTI via a plastic tube. The test was performed according to the Association Française de NORmalisation (AFNOR) standard parameters (25). Due to the vaporization of acetonitrile, the number of puffs per series was reduced to 5, and 8 series with a total of 40 puffs were performed. The time between each series was set to 150 ± 30 s to check the volume in each chamber of the GTI and refill the chambers if necessary (Figure A1).

### 2.5. Quantification of Beclomethasone Dipropionate by High-Pressure Liquid Chromatography–UV

The determination of the active substance mass (in the various GTI chambers and NGI stages) was carried out using HPLC (Shimadzu, Japan), a frequently used analytical method for the dosing of molecules in liquid form. The detection system used a UV detector (SPD-40D) to detect the entities that were analyzed using LabSolutions software (Version 5.6, Shimadzu, Kyoto, Japan). The column used was an Acclaim™ 300 C18 (50 × 4.6 mm, 3 µm, Thermo Scientific, Waltham, MA, USA) to perform reversed-phase chromatography. The wavelength was set to 248 nm, which corresponds to the maximum absorption wavelength of beclomethasone dipropionate. The mobile phase consisted of 70:30 acetonitrile:water (*v*/*v*) and was forced into the column with a pump (LC-40B XR) at a rate of 0.5 mL/min. The column was placed in an oven (CTO-40C, Shimadzu) to heat the samples to 25 °C. The autosampler (SIL-40C, Shimadzu) was set to 10 µL per injection. The tests were performed in triplicate, and each sample was injected twice to test repeatability.

The results were then interpreted using a calibration curve previously generated with increasing concentrations of beclomethasone dipropionate in acetonitrile (1; 5; 10; 25; 50 µg/mL; y = 8076.1x − 4734 and R^2^ = 0.9939).

The linearity of the calibration curve with linear regression coefficient of determination (R^2^) > 0.990 and the absence of a matrix effect from PDO (1,3-propanediol), which does not absorb in the UV range, and Poloxamer 188, with a retention time different from that of the BDP, was checked in order to estimate the concentrations in our samples.

## 3. Results

### 3.1. Comparison of Respirable Dose Between the Nebulizer and VDDS

Nebulization of beclomethasone dipropionate with the jet nebulizer shows a respirable dose of 157.6 ± 47.6 µg (Table 2). This corresponds to 19.7 ± 5.9% of the nominal dose (800 µg) that was filled into Cirrus™ 2. This indicates that a large proportion of the drug is lost during the nebulization. Indeed, it is known that nebulizers have very poor efficiency to deliver a respirable dose of aerosol due to several phenomena such as aerosol exhalation or the recycling of large droplets, that remain in the liquid chamber of the nebulize as a dead [28,29].

**Figure 2:**
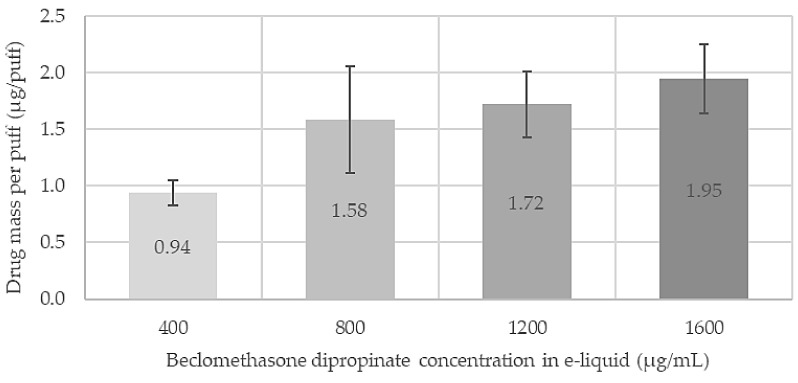
Respirable dose fraction of beclomethasone dipropionate divided by 40 puffs generated with VDDS for different concentrations of beclomethasone dipropionate e-liquid. Data are presented as mean ± standard deviation from three independent experiments.

The amount of active ingredient delivered in a single puff of the VDDS at different concentrations of beclomethasone dipropionate in the e-liquid is presented in Figure 2. Data are presented as the mean ± standard deviation from three independent experiments. The plot shows the drug mass per puff calculated by dividing the total respirable dose delivered by the VDDS by the number of puffs performed (40 puffs in these specific experimental conditions). Interestingly, the drug mass per puff almost doubles (0.94 ± 0.11 to 1.58 ± 0.47 µg/puff) when the drug concentration in the e-liquid increases from 400 to 800 µg/mL. At higher concentrations, the mass of drug per puff begins to stagnate, increasing only slightly from 1.72 ± 0.29 to 1.95 ± 0.31 µg/puff at the initial drug concentration in the e-liquid of 1200 and 1600 µg/mL, respectively. Overall, these results showed that the VDDS was able to deliver quantifiable and increasing respirable doses of beclomethasone dipropionate depending on the initial drug concentration in the e-liquid.

### 3.2. Comparison of the Duration of the Administration of the Aerosol Between the Nebulizer and VDDS

The comparison of the duration of administration is shown in Table 2.

The average duration of nebulization was 14 min 24 s ± 28 s. To determine the time required to administer the same dose by the VDDS, the number of puffs required to match the average dose of beclomethasone dipropionate generated by nebulization was first determined for each concentration by dividing the total respirable dose obtained with nebulization by the respirable dose contained in a single puff. The numbers of puff required ranged from 168 (400 µg/mL) to 81 puffs (1600 µg/mL). Then, two durations of beclomethasone dipropionate administration using VDDS puffing were calculated. The aerosol duration scenario was solely based on the recommended puff duration of 3 s while the patient administration duration scenario added an inter-puff interval of 30 s as recommended in the AFNOR standard XP-90-300-3 [30]. The scenario with patient administration duration is therefore closer to a realistic use of a VDDS by a patient than the aerosol duration scenario. As expected, the duration of administration of the drug by the VDDS decreased with increasing drug concentration in the e-liquid to reach the dose corresponding to that of the nebulizer in both scenarios. The aerosol duration scenario ranged from 8 min 24 s to 4 min 03 s depending on the initial drug concentration of the e-liquid and was shorter than the total nebulization time of approximately 15 min. However, in the more “realistic” patient administration duration scenario for the patient, the administration times increased to approximately 90 min (400 µg/mL concentration) and 45 min (1600 µg/mL concentration) to achieve a respirable dose equivalent to that of a nebulizer. Finally, the optimal parameters of beclomethasone dipropionate administration by the VDDS were achieved with 81 puffs aerosolized from a 1600 µg/mL beclomethasone dipropionate e-liquid concentration compared to nebulization, which corresponds to an aerosol duration of about 4 min (i.e., four times lower than the aerosol duration compared to a jet nebulizer of about 15 min) and a patient administration time of about 45 min (i.e., three times higher than the patient administration duration compared to a jet nebulizer of about 15 min).

### 3.3. Comparison of the Mass Median Aerodynamic Diameter (MMAD) Between the Nebulizer and VDDS

**Figure 3:**
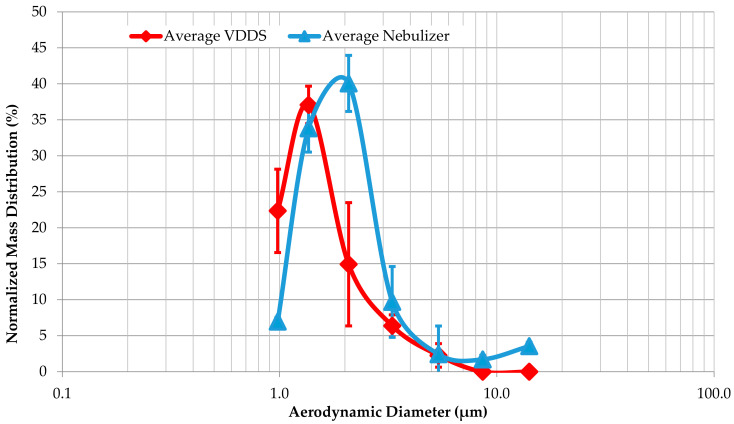
Comparison of normalized mass distribution between vaping drug delivery system (VDDS) and nebulizer. Data are presented as mean ± standard deviation for each point from three independent experiments.

**Figure 4:**
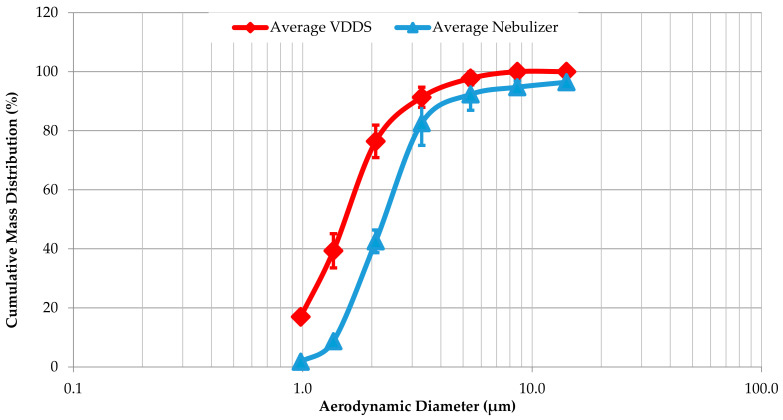
Comparison of cumulative mass distribution between vaping drug delivery system (VDDS) and nebulizer. Data are presented as mean ± standard deviation for each point from three independent experiments.

The GS-Tank produces beclomethasone dipropionate particles with an MMAD of 1.56 ± 0.05 µm and a GSD of 1.75 ± 0.23, while the particles from the Cirrus™ 2 have an MMAD of 2.30 ± 0.19 µm and a GSD of 1.64 ± 0.10 (Figure 3). According to Student’s t-test performed with Excel (Version 2409), the *p*-value of 0.006 indicates a significant difference between the MMAD, with a confidence interval of 95%. As we can see from the cumulative mass distribution, the VDDS also produces 20% of the particles with a diameter less than 0.98 µm (Figure 4). The nebulizer also produces large particles with an aerodynamic diameter of more than 10 µm and deposition in the throat.

## 4. Discussion

The use of a VDDS as a substitute for medical devices for the administration of inhalation therapies is still in its infancy, and very little data are available. Furthermore, most studies focused on bronchodilator administration, and only two studies were based on corticosteroid suspension or nanosuspension [17,21]. In this study, we investigated the feasibility of administering beclomethasone dipropionate with a high-power VDDS compared to a clinical jet nebulizer in terms of respirable dose, aerosol size distribution, and duration of administration.

One key parameter for the use of a VDDS in the administration of inhaled therapies is the thermal stability of the drug candidate. Since a VDDS is a heating device, the boiling point of the drug must be lower than the heating temperature of the VDDS and the boiling point of the solvent. In a previous study, Chaoui et al. failed to detect ipratropium bromide in the VDDS aerosol because its boiling point (230 °C) was close to that of the PDO solvent (210 °C), resulting in a transition and loss of the drug in the vapor state [20]. The boiling point of beclomethasone dipropionate is approximately 630 °C, which is higher than the boiling point of the PDO solvent used in this study. Therefore, beclomethasone dipropionate did not transfer into the vapor phase so that it could be quantified in the particle phase of the VDDS aerosol. Furthermore, the thermal stability of beclomethasone dipropionate appeared to be high enough given the relatively high VDDS power (30 W) used in this work. Indeed, in a previous study, salbutamol hemisulfate was found to be extremely sensitive to heat and was not quantifiable in aerosols generated with a VDDS with a power of only 15 W [20]. Therefore, beclomethasone dipropionate could be considered a potent drug candidate regarding its physicochemical properties.

Due to the poor solubility of corticosteroids in water, the e-liquids were prepared with stabilizers to increase their solubility. The choice of poloxamer 188 as a surfactant and stabilizer for the formulation was motivated by an existing study [17], but there are different types of surfactants that can be used [31]. On the other hand, Casula et al. showed in a previous study that a preparation of freeze-dried beclomethasone dipropionate nanocrystals significantly increased the solubility of beclomethasone dipropionate compared to raw powder and a mixture with poloxamer 188, resulting in twice the concentration of beclomethasone dipropionate in the particulate phase of the aerosol [17]. This study with freeze-dried nanocrystals of beclomethasone dipropionate interestingly showed smaller particles in the aerosol produced by the electronic cigarette, compared with those in our study, with an average diameter of 211 nm. Since the deposition of particles in the airways depends on the size of the particles, a nanosuspension could increase the efficiency in the treatment of CRD [32]. In addition, there are several other nano-delivery systems that could be tested to find the optimal combination for a VDDS and poorly water-soluble molecules. Therefore, optimization of the formulation with different excipients and nanoparticle sizes needs to be investigated to increase the concentration of beclomethasone dipropionate in the e-liquid to generate a higher delivered respirable dose and so reduce the number of puffs required to achieve an equivalent dose to a nebulizer.

Regarding the respirable dose, we found a positive correlation between the initial beclomethasone dipropionate concentration in the e-liquid and the respirable dose emitted by the VDDS. The nicotine concentration in the e-cigarette aerosol was shown to increase linearly with the nicotine concentration in the e-liquid. Interestingly, in a previous study, the inhalable dose of terbutaline started to reach a ceiling at an initial concentration of 1 mg/mL in the e-liquid [18]. In contrast, the respirable dose of beclomethasone dipropionate measured in our study continued to increase from an initial concentration in the e-liquid of 1.2 mg/mL. Despite the use of identical power settings (30 W and 1.5 Ω resistance), the respirable dose of beclomethasone dipropionate (≈1.95 µg/puff) was about ten times lower than the values reported for terbutaline (≈20 µg/puff) in previous studies [19,20]. This could be explained by the use of a different atomizer (GS-Tank in this study). Indeed, the design of the atomizer has been shown to play a key role in the respirable dose for an identical initial drug concentration and VDDS power level and could be used as an effective lever to optimize beclomethasone dipropionate delivery [19]. In addition to the design of the nebulizer, the power of the VDDS is another key parameter for drug delivery. Increasing the power from 20 W to 30 W led to a threefold increase in the delivered dose of salbutamol, while setting the power to 40 W did not further increase the delivered dose in a previous study [22]. The stabilization of the released dose beyond a power threshold was also observed for terbutaline, which increased linearly from 10 W to 30 W and stabilized over 30 W [16,18]. Therefore, the choice of 30 W in this work was consistent with the previous findings mentioned above but may not be entirely optimal as both the design of the nebulizer and the power affect the respirable dose of drug delivered by a VDDS [19]. Finally, care should be taken when increasing the power of the VDDS, as this could exponentially increase the generation of hazardous volatile organic compounds beyond 40 W [33]. Nevertheless, the solvent (PDO) used in our work is considered a GRAS (Generally Recognized as Safe) compound, which is recognized by the Food and Drug Administration in the food industry and has a lower thermal decomposition than propylene glycol (PG) [26].

In addition to a sufficient respirable dose, the VVDS must be able to generate particles that can reach the deep lung to adequately target the pathological areas. The optimal MMAD for inhaled therapies is usually between 1 µm and 5 µm [34]. We found that the average MMAD of the particles produced by the VDDS was 1.56 µm, which was in the optimal range, while the nebulizer produced larger particles with an MMAD of about 2.30 µm. Interestingly, the VDDS also produced a population of submicron particles that were not found with the nebulizer. These submicron particles offer several advantages for drug delivery, especially deep targeting ability [35]. Conversely, the nebulizer also produces a fraction of particles with an MMAD greater than 10 µm, which do not penetrate the lungs and are lost in the oral cavity [32,34]. The loss of larger particles—and the drug—in the oral cavity, combined with low patient compliance with medical devices for inhaled therapies [36], and the ability of a VDDS to produce submicron particles rationalize the use of a VDDS for the administration of inhaled drugs, even if the administration time is longer than with nebulization [36].

Overall, the VDDS has shown that it is possible to generate a beclomethasone dipropionate aerosol in vitro with similar performance (MMAD and respirable dose) to a jet nebulizer. The duration of nebulization to achieve the same amount of efficient drug mass is comparable to nebulization. However, the two durations calculated in this study may represent the extreme durations due to the unrealistic conditions of inhalation behavior. In fact, patients do not inhale without a pause, nor do they always take 30 s between puffs. This could lead to variations in nebulization duration depending on the patient’s behavior.

Regarding their use, VDDSs as new medical devices could bring many advantages for the effective treatment of CRDs. Conversely, VDDSs are smaller and more portable than nebulizers, making them more convenient for treatment outside the home [34]. Another advantage of VDDSs versus the other medical devices on the market is the ease of use. Different generations of vaping devices exist, but their mechanism to produce the aerosol are quite similar. The patient only has to press a button to draw the aerosol or can directly draw the aerosol without any coordination ability [14,37]. In fact, the lack of coordination or knowledge among 50% of patients is responsible for the misuse of medical devices such as PMDI and induces poor efficiency in CRD treatment [38]. Another dimension is the possible customizable and controlled dose delivered to the patient by only changing the wattage on the device settings [18,22]. Personalized medicine centers the treatment around the patient to adapt it to the requirement of the situation. This urges the reinforcement of the knowledge of patients regarding their healthcare and potentially increases the efficiency of the treatment for long-term diseases. These benefits of a VDDS could lead to an increase in the adherence of patients to their chronic therapy and bring better results by improving the quality of life [34]. As the use of VDDSs for inhaled drug delivery is still an emerging area of research, many challenges remain to be overcome. One of the main limitations is the thermal degradation of the drug, which could potentially be addressed by the development of low-power devices that would also limit the production of harmful VOCs. However, it should be noted that a low-powered device may reduce the quantity of the aerosolized drug, resulting in a higher number of puffs required to achieve the dose equivalent to that of a nebulizer. This limitation could be mitigated by encapsulating drugs in micro- or nanocarriers to enhance their thermal stability [39]. The efficiency of a VDDS to deliver a therapeutic dose may also be questionable, as 81 puffs were required to reach the dose equivalent to nebulized beclomethasone dipropionate in our study. Interestingly, the use of a salt-free form of salbutamol yielded higher emitted doses for VDDS delivery compared to salbutamol sulfate in a previous study [22], resulting in a total emitted dose equivalent to that of a marketed salbutamol inhaler in a single puff. Salt-free formulations are therefore potential candidates for VDDSs at the cost of a lower stability compared to the salt form. Finally, better control of the wattage output could allow future VDDSs to fine-tune the amount of drug released and the MMAD to adapt to the needs of patients according to their pathology and the severity of airway obstruction.

## 5. Conclusions

The aim of this study was to investigate in vitro whether the use of vaping technology can challenge the current delivery system in terms of duration, respirable dose, and particle size distribution. Varying the e-liquid concentration from 400 µg/mL to 1600 µg/mL showed the positive tendency of this factor on the drug mass contained in a single puff, from 0.94 µg to 1.95 µg, respectively. At a high concentration of beclomethasone dipropionate in the e-liquid, a number of 81 breaths was reached to achieve the same respirable dose as with nebulization. The determination of the required equivalent duration showed variability in the administration duration due to the vaping puffing behavior, corresponding to the consideration of the aerosol duration of 4 min (better than 15 min of nebulization) or the administration duration of 45 min (worse than the nebulization duration). In terms of particle size distribution, the VDDS was found to be able to produce smaller particles than the nebulizer (1.56 ± 0.05 µm vs. 2.30 ± 0.19 µm), which is beneficial for deeper lung deposition in lung disease. These results indicate that a VDDS is a potential alternative to the nebulization of beclomethasone dipropionate, suitable for the daily treatment of CRDs. The ease and convenience of use could promote patient adherence to improve the poor outcomes of current chronic therapies for CRDs.

## Figures and Tables

**Table 1:** Quantity of material for formulation of homemade e-liquid (87.5:12.5 PDO:stock suspension (*v*/*v*)).

E-Liquid Concentration (mg/mL)	0.4	0.8	1.2	1.6
PDO:stock suspension (*v*/*v*)	87.5:12.5	87.5:12.5	87.5:12.5	87.5:12.5
E-liquid volume (mL)	10	10	10	10
Stock suspension/deionized water volume (mL) (12.5%)	1.25	1.25	1.25	1.25
PDO volume (mL) (87.5%)	8.75	8.75	8.75	8.75
Stock solution concentration (mg/mL)	3.2	N/A	N/A	N/A
Stock solution volume (mL)	5	N/A	N/A	N/A
Beclomethasone dipropionate mass (mg)	16	8	12	16
Poloxamer 188 mass (mg)	8	4	6	8

**Table 2:** Comparison of duration between nebulization and vaping drug delivery system (VDDS) at different concentrations for equivalent respirable dose of beclomethasone dipropionate with two inhalation conditions.

Nebulizer	VDDS			
400 µg/mL	400 µg/mL	800 µg/mL	1200 µg/mL	1600 µg/mL
Respirable dose: **157.6 µg/nebulization of 2 mL**	Respirable dose: **37.5 µg/40 puffs**	Respirable dose: **63.4 µg/40 puffs**	Respirable dose: **68.9 µg/40 puffs**	Respirable dose: **77.9 µg/40 puffs**
Nebulization time: **14 min 24 s ± 28 s**	**Equivalent respirable dose of beclomethasone dipropionate delivered by VDDS compared to nebulization**			
	Number of puffs: **168** Aerosol duration: **8 min 24 s** Patient administration duration: **91 min 55 s**	Number of puffs: **100** Aerosol duration: **4 min 59 s** Patient administration duration: **54 min 15 s**	Number of puffs: **92** Aerosol duration: **4 min 35 s** Patient administration duration: **49 min 52 s**	Number of puffs: **81** Aerosol duration: **4 min 03 s** Patient administration duration: **44 min 02 s**

## Data Availability

The data that support the findings of this study are available from the corresponding author upon reasonable request.
